# Rigid enlargement of sybodies with antibody fragments for cryo-EM analyses of small membrane proteins

**DOI:** 10.1038/s41598-025-92950-5

**Published:** 2025-03-19

**Authors:** Fabian Ackle, Sujani Thavarasah, Jennifer C. Earp, Markus A. Seeger

**Affiliations:** Institute of Medical Microbiology, University of Zurich, Zurich, Switzerland

**Keywords:** Cryo-EM, Fab, Nanobody, Sybody, Fiducial marker, Size enlargement, Particle alignment, Proteins, Structural biology

## Abstract

Single particle cryo-electron microscopy (cryo-EM) has become the method of choice to determine experimental structures of integral membrane proteins. However, high-resolution structure determination by cryo-EM remains a challenge for membrane proteins that are too small or lack distinctive structural elements for particle alignment. To address this problem, single-domain antibodies called nanobodies and their synthetic variants called sybodies are widely used tools to trap membrane transporters in defined conformations, to enlarge particle sizes and to act as fiducial markers enabling reliable particle alignment. Recently, antibody fragments (Fabs) enlarging nanobodies at their backside in a rigid fashion, called Legobody and NabFab, have been developed. Here, we investigated how Legobodies and NabFabs can be harmonized with sybodies. We show that any sybody can be adapted to the Legobody approach with minimal effort, while only a subset of sybodies belonging to the loop library can be converted into a format recognized by the NabFab without complementarity-determining region-grafting. This technical note will facilitate the usage of Legobodies and NabFabs in the context of sybodies targeting membrane proteins and other small proteins for high-resolution structure determination by cryo-EM.

## Introduction

Improvements in direct electron detectors have sped up and increased the sensitivity of data collection in cryo-EM. Together with advancements in software and an increase in computational power, these developments have led to the resolution revolution in cryo-EM^1^. Cryo-EM has become the preferred method for the experimental structure determination of large proteins and protein complexes, particularly membrane proteins, which were difficult or impossible to study using X-ray crystallography. However, it remains challenging to study smaller proteins with sizes below 100 kDa by cryo-EM, due to their small particle size, low contrast, or lack of distinguishable features for particle alignment and averaging^2^.

One approach to tackle the problem of investigating small membrane proteins by cryo-EM is to engineer small protein domains into loops of the protein of interest. For example, an engineered variant of the α-helical protein apocytochrome b562RIL (BRIL) can be introduced into the α-helices of membrane proteins, and the size of the fusion protein can be further increased by a BRIL-binding Fab fragment^3,4^. However, despite the stability of BRIL-fusion constructs, there is some flexibility at the fusion junction site^4^. The most prevalent approach to enlarge particle size is the use of affinity reagents, including Fabs and single-domain antibodies (sdAbs), also known as nanobodies.

Nanobodies have emerged as popular research tools to target membrane proteins. While nanobodies are traditionally obtained upon immunization of camelids^5^, they are increasingly generated entirely in vitro from synthetic nanobody libraries^6–8^. Sybodies, which are selected by a combination of ribosome display and phage display, represent one of these synthetic nanobodies^9^. Sybodies have been engineered to mimic the shape variability found in natural nanobodies and therefore are available in three sub-libraries called concave (short CDR3), loop (medium CDR3) and convex (long CDR3)^8,9^. While the concave and loop sybodies share the same scaffold, the convex sybodies are designed based on a different scaffold in order to stabilize CDR3.

Sybodies have been instrumental in successfully determining cryo-EM structures of challenging membrane proteins. For example, cryo-EM structures were obtained of the 32 kDa transporter LicB and the 43 kDa monomeric scramblase XKR9 after enlarging these proteins using sybodies^10,11^. A combination of using sybodies targeting *Neisseria gonorrhoeae* LptDE and further enlargement with maltose-binding protein (MBP) resulted in a high-resolution cryo-EM structure of this β-barrel protein^12^. Sybodies also increased the thermal stability of *Arabidopsis thaliana* PIN1 which was crucial for solving its structure^13^. Other examples include the secondary active transporter Glt_Tk_ of which a structure obtained in the presence of an inhibitory sybody shed light on the mechanism of aspartate uptake^14^. Similarly, cryo-EM structures of the volume-regulated ion channel LRRC8A in complex with inhibitory or activating sybodies, uncovered changes in the channel’s conformation, thereby providing information on the allosteric modulation of channel activity^15^. And finally, a sybody binding to the extracellular portion of the orexin receptor 2 was instrumental to obtain cryo-EM structures of this GPCR in its active, agonist-bound state^16^.

**Fig. 1:**
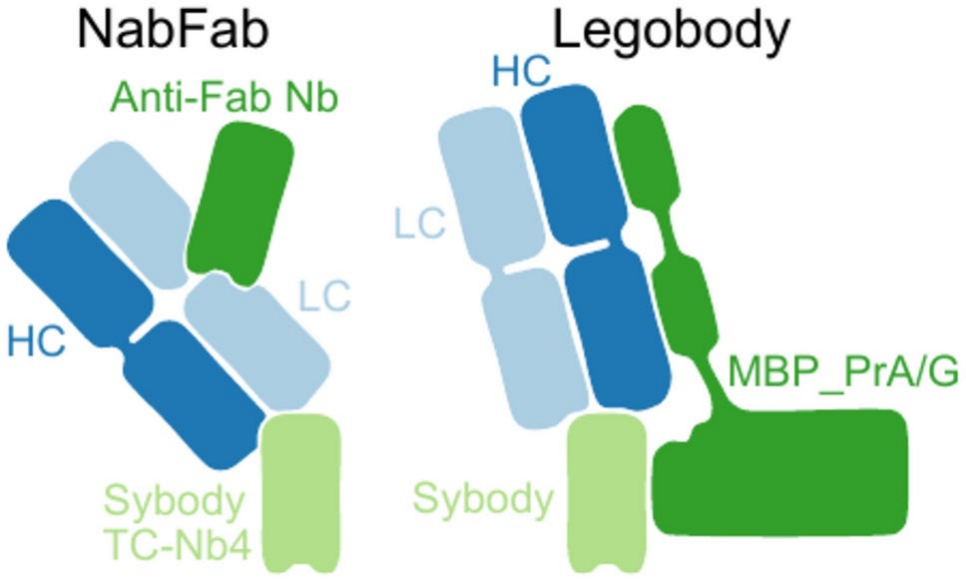
Scheme showing the NabFab and Legobody assemblies. In both, the Fabs (heavy chain, HC, in dark blue and light chain, LC, in light blue) bind to the backside of modified sybodies. For the NabFab assembly, the Fab binds to a sybody from the loop library (light green) harboring an amino acid change in its backbone or a sybody where the CDRs are grafted onto the TC-Nb4 scaffold. For the Legobody assembly, the Fab binds directly to sybodies (light green) containing an amino acid change in the backbone. The assemblies are completed with a Fab-binding nanobody (Anti-Fab Nb, green) in the case of NabFab, or with a sybody- and Fab-binding MBP_PrA/G fusion protein (green) in the case of Legobody.

A major drawback of nanobodies and sybodies is their moderate molecular weight (~ 15 kDa), which can be insufficient to enable structure determination of difficult small targets by cryo-EM. To address this issue, Fabs binding to nanobodies at their backside without disturbing nanobody-target interactions were developed^17,18^. In the Legobody approach^17^ (Fig. 1), a Fab was raised against a nanobody with high similarities to synthetic nanobody scaffolds created by McMahon et al*.*^6^ and Zimmermann et al*.*^8^. The Fab-nanobody complex was further enlarged by MBP fused to domain C of protein A through a shared helix, resulting in a rigid connection between MBP and the nanobody. This construct is further linked to two Fab-binding domains, protein G and domain D of protein A, collectively referred to as MBP_PrA/G. The resulting protein complex was called Legobody due to its resemblance to Lego construction. The utility of the Legobody was demonstrated by enlarging sybodies previously generated against the 25 kDa KDEL receptor^19^ and against the receptor-binding domain of SARS-CoV-2 spike protein (RBD)^20^, resulting in high-resolution cryo-EM maps with density sufficient for de novo model building and analysis of the binding interface to the respective sybody^17^. The Legobody approach was used by Kang et al*.* to solve the structure of the 21 kDa small uncoupling protein 1 utilizing a sybody selected against the protein^21^. Fan et al*.* determined the structure of the dopamine D_1_ receptor (DRD1) bound to LSD by fusing DRD1 to a modified β-arrestin-mimicking nanobody on which the Legobody complex was assembled^22^. Reimund et al*.* resolved the binding interfaces between apolipoprotein B100 (a structural component of low-density lipoprotein, LDL) and the LDL receptor. This was facilitated by the use of a Legobody, containing an LDL-binding nanobody to help with particle alignment and classification^23^. The second method, described by Bloch et al*.* involved the development of a tight nanobody-binding Fab called NabFab (Fig. 1)^18^. The NabFab was selected from a synthetic Fab library in vitro^24^ against an alpaca-derived nanobody called TC-Nb4, which binds to human transcobalamin and its cognate receptor TCblR/CD320^25^. An additional nanobody, which binds to the hinge region between the variable and constant domains of the Fab^26^, adds a distinctive element to the shape of the assembly. NabFabs can be used as a fiducial marker and size enhancer for high-resolution cryo-EM structure determination of small membrane proteins. This approach has been used to determine cryo-EM structures of VcNorM and ScaDMT^18^, and more recently an inward-facing structure of the bacterial melibiose transporter (MelB)^27^, the inactive-state histamine receptor 2 bound to famotidine^28^ and the μ-opioid receptor bound to a nanobody antagonist^29^.

In this work, we investigated how Legobodies and NabFabs can be attached onto the sybody scaffold. We found that sybodies can be easily converted into a format that is reliably recognized by Legobodies and with some exception also by NabFabs.

## Results

Previous structural analyses revealed that the Legobody Fab binds nanobodies distal to their CDRs, with binding interactions involving the C-terminal amino acids, including residues from the cloning scar, as well as parts of the His-tag (VTVSSLEHHHHHH, interfacing residues underlined)^17^. The sybody selection pipeline uses FX-cloning^30^ for subcloning of the binder library from the phage display vector into the expression vector pSBinit to avoid PCR amplification and its associated effects like loop shuffling^31^. However, this cloning procedure introduces a C-terminal alanine cloning scar^9^, thereby turning the last serine of the nanobody or sybody scaffold (VTVSS) into an alanine (VTVSA). In addition, in pSBinit, the C-terminus of the sybody is first followed by a myc-tag sequence and then the His-tag. In a series of affinity determination experiments using grating-coupled interferometry (GCI), we tested the sequence requirement of sybodies at the C-terminus to be recognized by the Legobody Fab. To this end, we first purified the RBD-binding sybody Sb#15^20^ with the following sequences at the C-terminus (sybody framework underlined):

(i) VTVSAGRAGEQKLISEEDLNSAVDHHHHHH (pSBinit^9^)
(ii) VTVSALEHHHHHH (new construct)
(iii) VTVSASLEHHHHHH (new construct)
(iv) VTVSSLEHHHHHH (as used by Wu et al.^17^)

**Fig. 2:**
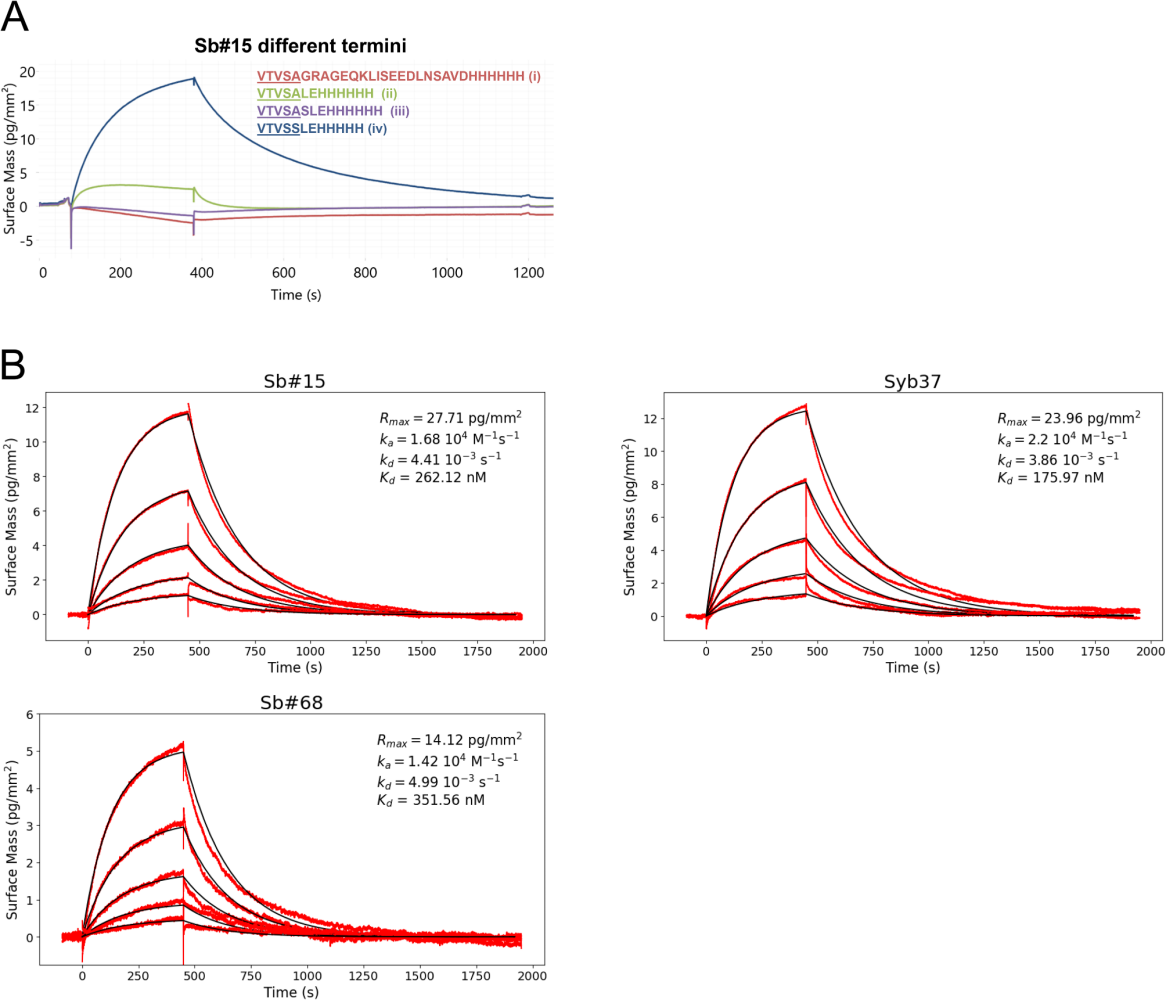
Sybody binding by the Legobody Fab. (**A**) Binding of sybodies with different termini by the Legobody Fab measured using GCI. (**B**) Affinity determination of the Legobody Fab binding to three sybodies stemming from three distinct sub-libraries (concave: Sb#15^20^, loop: Syb37^19^ and convex: Sb#68^20^). All these sybodies contain the C-terminus (iv) shown in (**A**). Measured data (red curve) was fitted with a Langmuir 1:1 binding model (black curve) and values for on-rate (k_a_), off-rate (k_d_) and dissociation constant (K_D_) are given in the respective graphs.

GCI analysis revealed that sybodies expressed from the pSBinit plasmid, the standard expression and screening vector of sybodies, were not bound by the Legobody Fab. Even when the motifs “LEHH” (construct ii) or “SLEHH” (construct iii) were introduced after the cloning scar alanine, no binding was observed. Only when the cloning scar alanine was reverted to a serine as present in the original construct used in the Legobody study^17^, the Legobody Fab could bind to the sybody (Fig. 2A).

To facilitate the use of sybodies with the Legobody approach, a new vector called pSBLego was constructed (available via Addgene #219967). The vector encodes a N-terminal PelB-signal sequence enabling periplasmic expression, and encodes for a C-terminal “SLEHHHHHH” motif. The vector is compatible with FX-cloning, but sybodies to be cloned into pSBLego need to be PCR-amplified with a new reverse primer (see methods) to replace the alanine cloning scar with a serine.

Next, we expressed representative sybodies belonging to the sybody sub-libraries^8^ concave (Sb#15, raised against SARS-CoV2 RBDs^20^), loop (Syb37, raised against KDEL receptor^19^) and convex (Sb#68, raised against SARS-CoV2 RBDs^20^) from pSBLego. GCI analysis revealed that these sybodies were all bound by Legobody Fab with K_*D*_ values ranging from 200 to 350 nM (Fig. 2B).

**Fig. 3:**
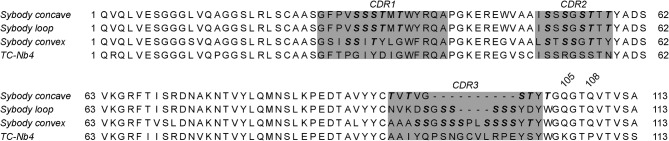
Protein sequence alignment of the three non-randomized scaffold sybodies and TC-Nb4, generated using Clustal Omega^37^ with Kabat numbering^39^. Gray boxes highlight the CDRs which are retained during grafting. Bold and italicized residues in the sybody sequences indicate randomized positions within the libraries.

**Fig. 4:**
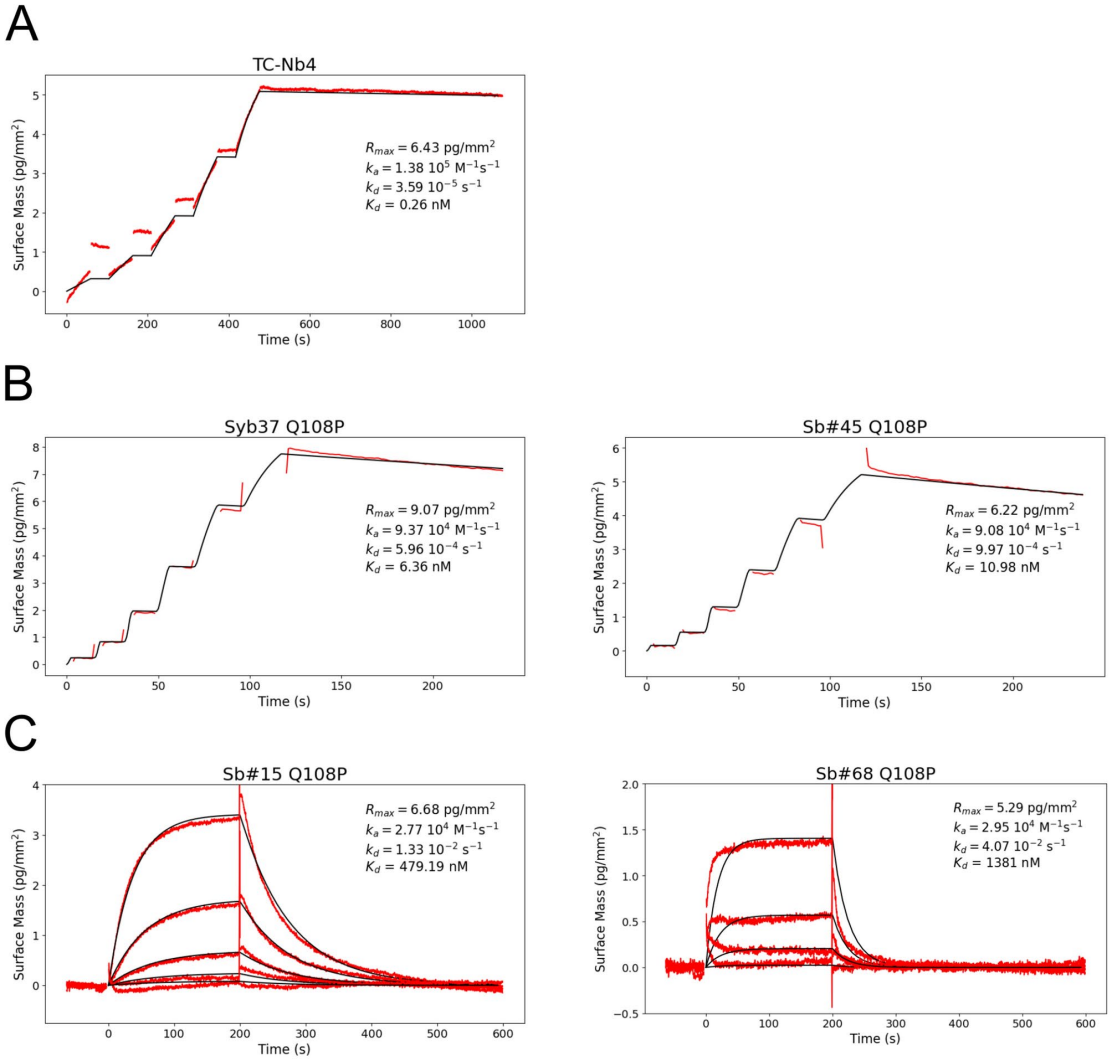
GCI measurements using NabFab. (**A**) Affinity determination of the NabFab against TC-Nb4. (**B**) Affinity determination of the NabFab against Syb37 Q108P and Sb#45 Q108P (both belonging to the loop library). (**C**) Affinity determination of the NabFab against Sb#15 Q108P (concave library) and Sb#68 Q108P (convex library). In all measurements, measured data (red curve) was fitted with a Langmuir 1:1 binding model (black curve) and values for on-rate (k_a_), off-rate (k_d_) and dissociation constant (K_D_) are given in the respective graphs.

The NabFab was generated through an in vitro selection using a synthetic Fab library, which was selected against TC-Nb4, an alpaca-derived nanobody against the complex of human transcobalamin and its cognate receptor TCblR/CD320^25^. TC-Nb4 differs in several framework amino acid residues from the sybody scaffold (Fig. 3). In particular, K105 and P108 present in TC-Nb4, which correspond to two glutamines in the sybody scaffold, appear to play an important role for binding interactions between the NabFab and the target nanobody, as remarked by Bloch et al*.*^18^. Based on our own analysis, we reasoned that especially the framework residue Q108 found in sybodies prevents NabFab binding due to a steric clash. We therefore introduced the amino acid change Q108P in a series of sybodies and tested binding to the NabFab using GCI. For the two loop library sybodies, Syb37 and Sb#45 (raised against SARS-CoV2 RBDs^20^), this substitution was indeed sufficient to restore tight NabFab binding, though with somewhat weaker affinity (K_*D*_ ≈ 10 nM) as compared to the original nanobody TC-Nb4 (K_*D*_ = 0.3 nM) (Fig. 4A, B). In contrast, only very weak NabFab binding was restored when the corresponding Q108P substitution was introduced into the concave sybody Sb#15 (K_*D*_ ≈ 500 nM) or the convex sybody Sb#68 (K_*D*_ = 1.4 μM) (Fig. 4C).

**Fig. 5:**
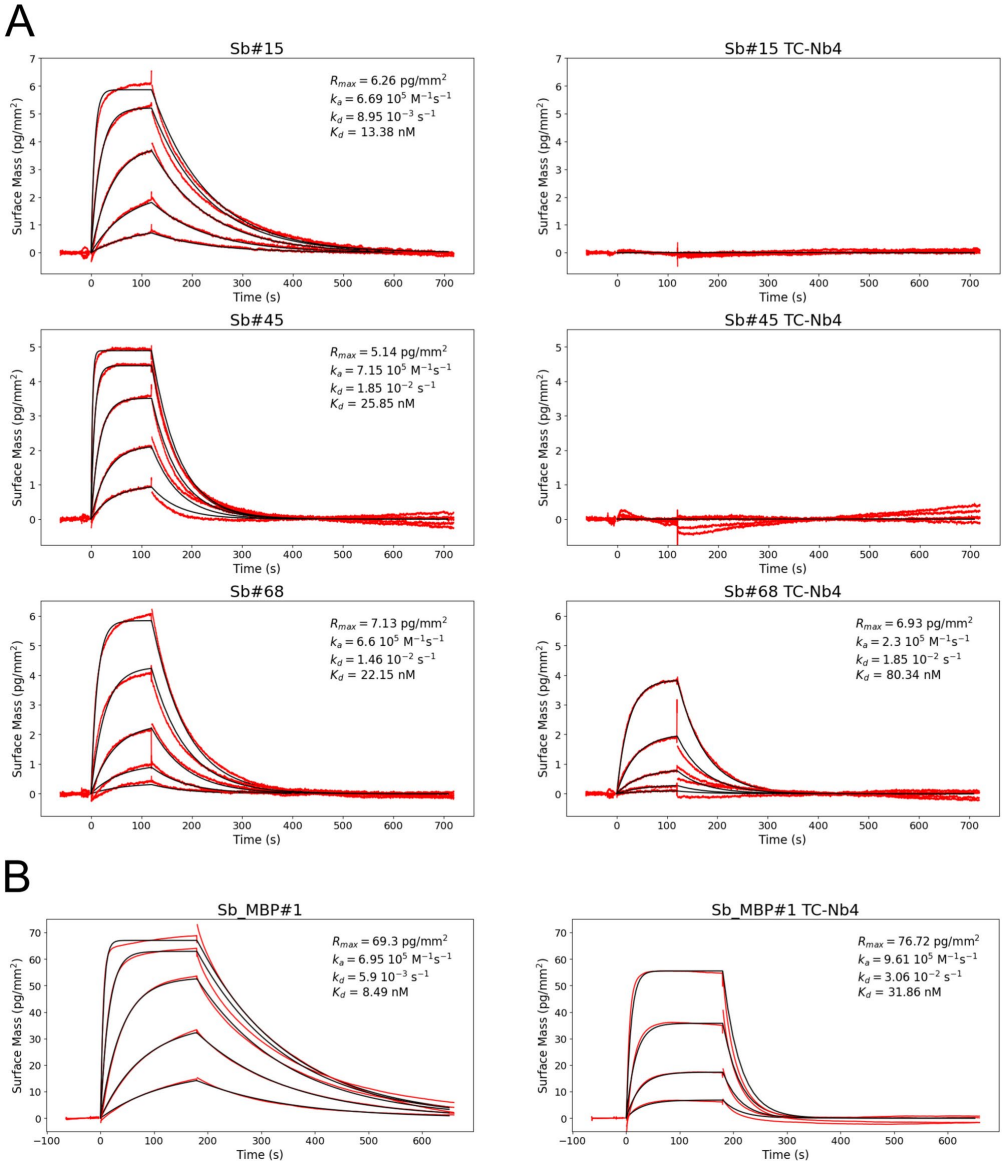
GCI measurement using loop-grafted sybodies. (**A**) Affinity determination of Sb#15, Sb#45 and Sb#68 and their grafted versions onto TC-Nb4 against immobilized SARS-CoV-2 spike protein. (**B**) Affinity determination of Sb_MBP#1 and its grafted version onto TC-Nb4 against immobilized MBP. Measured data (red curve) was fitted with a Langmuir 1:1 binding model (black curve) and values for on-rate (k_a_), off-rate (k_d_) and dissociation constant (K_D_) are given in the respective graphs.

As an alternative, we followed the CDR-grafting strategy outlined in Bloch et al*.*^18^. To this end, we grafted the CDRs of several sybodies onto the scaffold of TC-Nb4. To investigate whether CDR-grafting comes at the cost of target binding affinity, we performed GCI measurements to assess the affinity of the grafted sybodies for their respective target proteins. Sb#15, Sb#45 and Sb#68 are three SARS-CoV-2 RBD-specific sybodies stemming from the concave, loop and convex library, respectively^20^. While Sb#15 and Sb#45 exhibit high affinity to their targets with K_*D*_ values of 13 nM and 26 nM, respectively, they completely lost their affinity towards the SARS-CoV-2 RBD upon loop-grafting (Fig. 5A). In contrast, CDR-grafted Sb#68 is recognized by the NabFab (K_*D*_ = 80 nM), although at somewhat weaker affinity than the original sybody (K_*D*_ = 22 nM) (Fig. 5A). To assess whether convex sybodies are generally tolerant to grafting, the CDRs of sybody Sb_MBP#1 recognizing MBP of *E. coli* were grafted onto the TC-Nb4 scaffold. GCI measurements revealed that the binding affinity of grafted Sb_MBP#1 (K_*D*_ = 32 nM) is similar to the one of the original sybody (K_*D*_ = 8 nM) (Fig. 5B). Of note, in previously published surface plasmon resonance measurements, Sb_MBP#1 had a K_*D*_ = 22 nM^8^.

## Discussion

The in vitro selection of sybodies can yield high-affinity binders even against difficult targets such as labile proteins, membrane proteins or protein complexes. One of the key advantages is that sybody selections can be carried out in the presence of non-covalently bound ligands, thereby yielding conformation-specific binders. Sybodies and nanobodies have been shown to serve as fiducial markers, aiding in particle alignment and averaging by increasing particle size. The most prominent example is the cryo-EM structure of the 32 kDa transporter LicB, which was determined in complex with a sybody^11^.

However, in many instances the small size of a sybody or other small binding molecules such as a designed ankyrin repeat protein (DARPin) is limiting its capacity to improve cryo-EM structure determination. One reported size augmentation approach was to rigidly attach such small binding modules to the surface of large and symmetric macromolecular assemblies. For example, a DARPin was fused through a helical extension to a protein moiety which self-assembles into a cubic cage, finally displaying twelve DARPins at the cage surface^32^. In a recent study, a surface-exposed protein of adenovirus-derived nanoparticles was developed into a novel binding scaffold called ADDobody that can in principle be raised against any protein target to enable cryo-EM analyses^33^. Although not yet shown, it would be in principle possible to engineer a self-assembling cage that would display rigidly attached nanobodies or sybodies at their surface. However, the main challenge of the cage surface display approach is the rigidity of attachment; in the published example, the core of the cage was highly resolved in the cryo-EM maps, whereas densities typically become increasingly blurry the farther away the surface-binding module and its bound target stick out from the cage surface^32^.

An alternative approach is the rigid fusion of proteins to nanobodies. The first documented approach following this strategy was the megabody, which entails an enlargement of the nanobody scaffold via β-sheet extension using permutated HopQ or YgjK protein, thereby adding 45 kDa or 86 kDa of additional mass, respectively^34^. However, at least the HopQ megabodies exhibit a somewhat flexible hinge between nanobody and HopQ domain, effectively meaning that in cryo-EM reconstructions using the megabody approach, density for HopQ is blurred^35,36^. Another size augmentation strategy is to rigidly fuse MBP to the C-terminus of a sybody, an approach called Pro-macrobodies and used to determine the structure of *Neisseria gonorrhoeae* LptDE^12^.

Fabs recognizing the backside of nanobodies have been shown to largely overcome recurrent flexibility issues, as has been demonstrated by the Legobody and NabFab approach. However, in nanobodies derived from immune libraries, the backside is not entirely conserved, effectively meaning that Legobody and NabFab binding needs to be assessed either based on sequence alignments and/or experimental approaches.

In stark contrast, a key advantage of sybodies (and other synthetic nanobodies) is their conserved framework, which only in exceptional cases harbors (unintended) amino acid changes at their backside. In this work, we assessed how well NabFabs and Legobodies harmonize with sybodies, thereby providing simple guidelines to augment their size in an efficient manner.

We could show that if the residues directly following the C-terminus of sybodies are adequately adjusted by introducing the VTVSSLEHHHHHH motif, the Legobody Fab binds to the backside of any sybody with a dissociation constant between 200 and 350 nM. Hence, sybodies can easily be converted into a Legobody by subcloning the sybody into and expressing it from the newly designed pSBLego vector, followed by the addition of the Legobody Fab and MBP_PrA/G. Importantly, binding of MBP_PrA/G further reinforces the complex.

The NabFab approach works reliably for sybodies stemming from the loop library. An amino acid change (Q108P) needs to be introduced close to the C-terminus during subcloning into the expression vector to allow for NabFab binding. Binding affinities of NabFab to modified loop sybodies are in the range of 10–20 nM, which is somewhat weaker than to its cognate TC-Nb4 scaffold, but sufficiently strong for cryo-EM analyses. When the same approach of introducing the Q108P amino acid change was tested for sybodies of the concave or convex library, NabFab binding was not restored. In case of concave sybodies, lack of binding can be explained by the fact that W103, which directly follows the original CDR3 sequence and interacts with the NabFab, is randomized in the concave sybody library. For Sb#15, this results in a W103I substitution. In general, concave sybodies rarely carry a tryptophan in this position and thus are not easily made compatible for NabFab enlargement. In case of the convex sybodies, which deviate in several positions from the framework of the concave and loop sybodies, we suspect that L89, which in most of the nanobodies and in concave and loop sybodies is V89, could impede NabFab binding.

We also assessed grafting of sybody CDRs onto the TC-Nb4 scaffold, as has been proposed by Bloch et al*.*^18^ as a general approach to make any nanobody compatible with NabFabs. We found that CDRs grafted from two different sybodies stemming from the convex library onto the TC-Nb4 scaffold results in chimeric nanobodies, which retained binding affinity to the respective targets, namely SARS-CoV-2 RBD and MBP. Hence, we can recommend the grafting approach for convex sybodies. However, grafting of CDRs from concave and loop sybodies onto the TC-Nb4 scaffold resulted in chimeric molecules that lost their capacity to bind to their respective targets, although they could be purified without any problem.

As a further disclaimer, one should keep in mind that in some cases, the enlarged sybody complex might no longer bind to the target owing to steric clashes that might occur between Fab and the target protein. Wu et al*.*^17^ and Bloch et al*.*^18^ already provided a comprehensive steric clash analysis based on deposited structures of nanobody complexes in the PDB and found that clashes are expected to occur occasionally. To rule out clashes of enlarged sybodies, additional experiments such as size exclusion chromatography or ELISA need to be carried out.

**Fig. 6:**
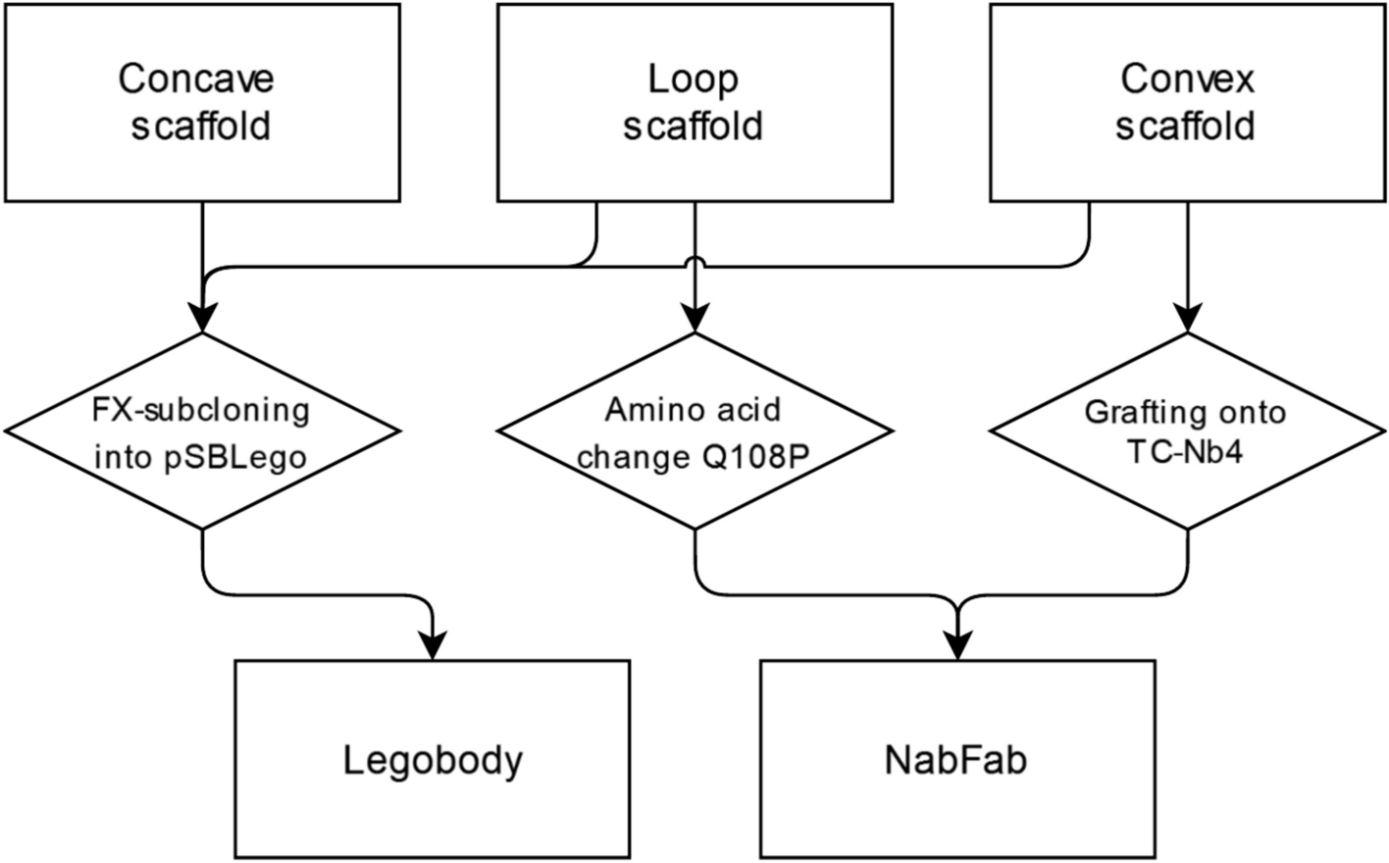
Flowchart showing the adaptation of the three sybody scaffolds for the Legobody and NabFab approach. Concave, loop and convex sybodies are adapted for the Legobody approach by FX-subcloning into the pSBLego vector. To achieve compatibility with the NabFab approach, loop sybodies are modified with the Q108P amino acid change through site-directed mutagenesis, and the CDRs of convex sybodies are grafted onto TC-Nb4.

For a general workflow, we suggest performing a sybody selection against the protein of interest, as this yields various binders with different affinities, binding epitopes and from different sybody sub-libraries. Sybodies alone may enable structure determination of the protein of interest^10,13,14^. Hence, we would recommend to first perform cryo-EM analyses of the more simple sybody complexes, thereby also assessing sybodies binding to different epitopes. If the sybodies are not sufficient as fiducial markers, they can be made compatible for Legobody or NabFab enlargement with minimal effort according to the strategy summarized in the flowchart below (Fig. 6). With these simple guidelines we hope to further facilitate the use of sybodies for cryo-EM analyses of small (membrane) proteins.

## Material and methods

### Construction of the pSBLego expression vector

pSBLego was cloned by PCR, phosphorylation and blunt-end ligation. First, pBXPC3H was linearized by PCR using the forward primer 5’-TGAGTTTAAACGGTCTCCAGCTTG and the reverse primer 5’-TGCAGAAGAGCTGAACTAGTGGATC, followed by column-purification of the PCR product. In a second PCR, the product from the first PCR was used as a template and the sequence encoding “SLEHHHHHH” was introduced with the forward primer 5’-TTGGAACACCATCACCACCACCATTGAGTTTAAACGGTCTCCAGCTTG and reverse primer 5’-GGAAGAAGAGCTGAACTAGTGGATCCC. To self-circularize the PCR product, the column-purified DNA was incubated with 1 µl T4 DNA ligase (Thermo Scientific), 0.5 µl T4 polynucleotide kinase (NEB) and 0.5 µl DpnI (NEB) in 20 µl 1X T4 DNA ligase buffer. *E. coli* DB3.1 cells (Invitrogen) were transformed with 2 µl of the ligation reaction mixture. The sequence of the resulting construct was confirmed by Sanger and Nanopore sequencing.

### Sybody subcloning into pSBLego

Sybody DNA was amplified by PCR from the sybody pSBinit constructs with the primer pair Med_FX_for 5’- ATATGCTCTTCTAGTCAGGTTCAGCTGGTTGAGAGCG and Med_lego_FX_rev 5’- TATAGCTCTTCAGGAGCTCACAGTCACTTGGGTACC for the concave and loop library sybodies, or Long_FX_for and Long_lego_FX_rev for convex library sybodies. The PCR product was column-purified and subcloned into pBXPlego by FX-cloning^30^.

The glutamine to proline amino acid change was introduced during the sybody amplification step using the following reverse primers Med_NF_Q2P_FX_rev 5’-TATAGCTCTTCAGGAGCTCACAGTCACCGGGGTACC for the concave and loop library sybodies, or Long_NF_Q2P_FX_rev 5’-TATAGCTCTTCAGGAAGAAACGGTAACCGGGGTGCCC for the convex library sybodies.

### Sybody CDR-grafting onto TC-Nb4

The sybody protein sequences were aligned to the sequence of TC-Nb4 with Clustal Omega^37^. CDRs of the sybodies were retained and the remaining part of the protein scaffold was exchanged with the respective amino acid sequences from TC-Nb4, as previously described by Bloch et al.^18^. The resulting chimeric gene was ordered from Twist Bioscience.

### Sybody and target protein expression and purification

Sybodies were expressed and purified as described by Zimmermann et al.^9^. Sybodies were either expressed in a medium scale (50 ml) or large scale (0.6 l), extracted from the periplasm and purified using Ni–NTA affinity chromatography, followed by size exclusion chromatography using a Sepax SRT-10C SEC100 column (Sepax) equilibrated in TBS. The purified sybodies were stored at 4 °C. SARS-CoV-2 spike protein was expressed and purified as described by Walter et al.^20^. MBP was expressed and purified as described by Zimmermann et al.^8^.

### Legobody Fab expression and purification

The Legobody Fab expression vector was kindly gifted by Xudong Wu and Tom Rapoport. 100 ml Expi293F cells were transiently transfected using Expifectamine 293 transfection kit (Gibco) according to the manufacturer’s protocol and the expression was continued for three days. The supernatant was collected and imidazole was added to a concentration of 15 mM, followed by incubation with 12 ml of equilibrated Ni–NTA slurry (in PBS with 15 mM imidazole) for 30 min and loading onto a column. The resin was washed with 140 ml wash buffer (PBS with 50 mM imidazole) and the Fab was eluted with 10 ml elution buffer (PBS with 300 mM imidazole).

### MBP_PrA/G expression and purification

The MBP_PrA/G expression vector was kindly gifted by Xudong Wu and Tom Rapoport. The MBP_PrA/G fusion protein was expressed and purified according to Wu et al.^17^

### Protein biotinylation

The Fabs and SARS-CoV-2 spike protein were chemically biotinylated with EZ-Link NHS-Biotin (Thermo Scientific) according to the manufacturer’s instructions. MBP was enzymatically biotinylated as described by Kuhn et al.^38^. Purified NabFab was kindly gifted by Joël Bloch and Kaspar Locher.

### Grating-coupled interferometry (GCI)

Kinetic characterization of the sybodies and the two Fabs was performed using grating-coupled interferometry (GCI) on the WAVEsystem (Malvern Panalytical). All the GCI characterizations were performed at 25 °C in 20 mM Tris pH 7.5, 150 mM NaCl supplemented with 0.05% Tween-20. A Langmuir 1:1 model was used for data fitting.

For the screening of the Legobody Fab binding to sybodies with different termini, biotinylated Legbody Fab (40 μg/ml) was captured onto a PCP-STA WAVEchip (Malvern Panalytical) at a density of 1000 pg/mm^2^. One channel was left blank as a reference lane. Sb#15 with the termini “SLEHHHHHH”, “AGRAGEQKLISEEDLNSAVDHHHHHH”, “ALEHHHHHH” and “ASLEHHHHHH” were injected at a concentration of 200 nM over the two channels at a flow rate of 50 µl/min. The complex was allowed to associate and dissociate for 300 s and 1800 s, respectively.

To measure the binding kinetics of the Legobody Fab binding sybodies of the three different scaffolds, biotinylated Legbody Fab (40 μg/ml) was captured onto a PCP-STA WAVEchip at a density of 700 pg/mm^2^. One channel was left blank as a reference lane. Sb#15, Syb37 and Sb#68 were injected at concentrations of 200 nM, 100 nM, 50 nM, 25 nM and 12.5 nM over the two channels at a flow rate of 50 µl/min. The complex was allowed to associate for 450 s and dissociate for 1500 s.

To determine the binding kinetics of the NabFab to the TC-Nb4 scaffold, biotinylated NabFab (40 μg/ml) was captured onto a PCP-STA WAVEchip at a density of 750 pg/mm^2^. One channel was left blank as a reference lane. The TC-Nb4 binding kinetics were measured by regeneration-free kinetics, with a flow rate of 50 µl/min, injecting TC-Nb4 at concentrations of 100 nM, 50 nM, 25 nM, 12.5 nM and 6.25 nM. The complex was allowed to associate for 60 s and dissociate for 45 s, except for the highest concentration injection, which was allowed to associate for 60 s and to dissociate for 600 s.

To determine the binding kinetics of the NabFab to the modified loop sybody scaffold (Syb37 Q108P and Sb#45 Q108P), biotinylated NabFab (40 μg/ml) was captured onto a PCP-STA WAVEchip at a density of 1000 pg/mm^2^. One channel was left blank as a reference lane. The Syb37 and Sb#45 binding kinetics were measured by rapid kinetics with the tight binder configuration, and at a flow rate of 50 µl/min. Both sybodies were injected at 500 nM. The complex was allowed to associate for 120 s and to dissociate for 1800 s.

To determine the binding kinetics of the NabFab to the modified concave (Sb#15 Q108P) and convex sybody scaffold (Sb#68 Q108P), biotinylated NabFab (40 μg/ml) was captured onto a PCP-STA WAVEchip at a density of 1000 pg/mm^2^. One channel was left blank as a reference lane. Both sybodies were injected at 500 nM, 166.7 nM, 55.6 nM, 18.5 nM and 6.2 nM over the two channels at a flow rate of 50 µl/min. The complex was allowed to associate for 200 s and to dissociate for 400 s.

To determine the binding kinetics of the modified and grafted sybodies to the SARS-CoV-2 spike protein, biotinylated SARS-CoV-2 spike protein (40 μg/ml) was captured onto a PCP-STA WAVEchip at a density of 150 pg/mm^2^. One channel was left blank as a reference lane. The sybodies were injected in random order. Sb#15 and Sb#15-TC-Nb4 were injected at 200 nM, 66.7 nM, 22.2 nM and 7.4 nM, Sb#45 and Sb#45-TC-Nb4 were injected at 500 nM, 166.7 nM, 55.6 nM, 18.5 nM and 6.2 nM, Sb#68 and Sb#68-TC-Nb4 were injected at 100 nM, 33.3 nM, 11.1 nM, 3.7 nM and 1.2 nM. The complex was allowed to associate for 120 s and to dissociate for 600 s.

To measure the binding kinetics of Sb_MBP#1 and Sb_MBP#1-TC-Nb4 scaffold, biotinylated MBP (50 μg/ml) was captured onto a PCP-STA WAVEchip at a density of 450 pg/mm^2^. One channel was left blank as a reference lane. The sybodies were injected in random order at concentrations of 250 nM, 83.3 nM, 27.8 nM, 9.3 nM and 3.1 nM. The complex was allowed to associate for 180 s and to dissociate for 480 s.

To determine the binding kinetics of the NabFab to unmodified sybodies (Syb37 Q108P and Sb#45 Q108P), biotinylated NabFab (40 μg/ml) was captured onto a PCP-STA WAVEchip at a density of 1000 pg/mm^2^. One channel was left blank as a reference lane. Sybodies were injected at a concentration of 100 nM over the two channels at a flow rate of 50 µl/min. The complex was allowed to associate and dissociate for 200 s and 400 s, respectively.

## Data Availability

The pSBLego vector is available via Addgene under the Plasmid #219967 and the datasets used and/or analysed in the current study are available from the corresponding author on reasonable request.
